# A Glycolysis-Based Long Non-coding RNA Signature Accurately Predicts Prognosis in Renal Carcinoma Patients

**DOI:** 10.3389/fgene.2021.638980

**Published:** 2021-04-01

**Authors:** Honghao Cao, Hang Tong, Junlong Zhu, Chenchen Xie, Zijia Qin, Tinghao Li, Xudong Liu, Weiyang He

**Affiliations:** ^1^Department of Urology, The First Affiliated Hospital of Chongqing Medical University, Chongqing, China; ^2^Department of Urology, Rongchang Traditional Chinese Medicine Hospital, Chongqing, China; ^3^Central Laboratory, The First Affiliated Hospital of Chongqing Medical University, Chongqing, China

**Keywords:** glycolysis, lncRNA, prognosis, carcinoma, renal cell

## Abstract

**Background:**

The prognosis of renal cell carcinoma (RCC) varies greatly among different risk groups, and the traditional indicators have limited effect in the identification of risk grade in patients with RCC. The purpose of our study is to explore a glycolysis-based long non-coding RNAs (lncRNAs) signature and verify its potential clinical significance in prognostic prediction of RCC patients.

**Methods:**

In this study, RNA data and clinical information were downloaded from The Cancer Genome Atlas (TCGA) database. Univariate and multivariate cox regression displayed six significantly related lncRNAs (AC124854.1, AC078778.1, EMX2OS, DLGAP1-AS2, AC084876.1, and AC026401.3) which were utilized in construction of risk score by a formula. The accuracy of risk score was verified by a series of statistical methods such as receiver operating characteristic (ROC) curves, nomogram and Kaplan-Meier curves. Its potential clinical significance was excavated by gene enrichment analysis.

**Results:**

Kaplan-Meier curves and ROC curves showed reliability of the risk score to predict the prognosis of RCC patients. Stratification analysis indicated that the risk score was independent predictor compare to other traditional clinical parameters. The clinical nomogram showed highly rigorous with index of 0.73 and precisely predicted 1-, 3-, and 5-year survival time of RCC patients. Kyoto Encyclopedia of Genes and Genomes (KEGG) and Gene set enrichment analysis (GSEA) depicted the top ten correlated pathways in both high-risk group and low-risk group. There are 6 lncRNAs and 25 related mRNAs including 36 lncRNA-mRNA links in lncRNA-mRNA co-expression network.

**Conclusion:**

This research demonstrated that glycolysis-based lncRNAs possessed an important value in survival prediction of RCC patients, which would be a potential target for future treatment.

## Introduction

Renal cell carcinoma (RCC) is one of the most aggressive urinary system tumor, which accounts for nearly 4% of adult malignancies (Zhai et al., 2019). The latest statistical researches show that there are 403,262 new cases and 175,098 deaths in 2018 (Khadirnaikar et al., 2019). Histologically, there are many subtypes of RCC, which can be used to evaluate the prognosis. Despite significant advances in its diagnosis and treatment, the prognosis of RCC patients remains poor (Cheng et al., 2019). Due to the low sensitivity to radiotherapy and chemotherapy, the treatment of RCC is a systematic treatment based on radical resection (Heo et al., 2020). Recently, targeted therapy for RCC has been used in clinical cases, but the overall survival (OS) of patients is not as good as expected, especially for some terminal stage patients. Therefore, it is urgent to find the molecular atypia of RCC in order to distinguish between high- and low-risk patients, so as to facilitate early diagnosis and improve prognosis.

Long non-coding RNAs (lncRNAs) are the member of the non-coding RNA family, which have over 200 nucleotides in length (Michal et al., 2020). Previous genome sequencing studies have explicated that lncRNAs do not have the function of transcribing proteins, but play a vitally important role in variety of physiologic activities (Zhang Y. et al., 2020). Several studies have explicitly pointed out that lncRNAs participate in important cellular biological functions for their contributions in regulation of transcription and organization of nuclear domains (Yang et al., 2020). Meanwhile, lncRNAs play crucial roles in many cellular processes, which include glycolysis, cell differentiation and DNA repair (Wang Y. et al., 2019). According to these findings, abnormal expression of lncRNAs in tumor cell reveals that lncRNAs may be as potential bio-markers in survival prediction of RCC patients. Additionally, latest researches have revealed relation between non-mutational regulation of gene expression and drug resistance, during which lncRNAs could affect drug sensitivity to tumor cells as the major modulators (Xu et al., 2020). Based on the highly specific subtype of tumor cell, lncRNAs have been considered to have the potential ability to predict prognosis and provide novel therapeutic options.

The occurrence and migration of RCC have been verified to be related to glycolysis (Zhang C. et al., 2020). Glycolysis is a normal physiological metabolic process of human cells, which provides limited energy for the body. However, Glycolysis, known as the “Warburg effect,” is extremely active in tumor cell metabolism which decompose glucose or glycogen into pyruvate or lactic acid, even under aerobic environment (Chen et al., 2019; Kim et al., 2020). The glycolysis can product sufficient energy to tumor cell and inhibits apoptosis which promote proliferation and migration of cancer cell under severe environment (Wang S. et al., 2019). Interestingly, different solid tumors show different heterogeneity of glycolysis, such as breast cancer and non-small cell lung cancer (Guo et al., 2020; Smolle et al., 2020). Several studies have demonstrated that glycolysis can be used as a biological metabolic marker to predict the prognosis of tumors and further study of the mechanism of glycolysis can explore new targets to guide treatment.

Several studies have confirmed that the glycolysis-based lncRNAs affect solid tumor cells (such as colon cancer, breast cancer, and ovarian cancer) through epigenetic regulation (Chen et al., 2019; Lin et al., 2020; Zhang Z. et al., 2020). The role of the glycolysis-based lncRNAs in RCC is still unclear. In our study, we hypothesis that there are several lncRNAs related to glycolysis genes and may contribute to predict prognosis of RCC patients. By combining the clinicopathological types and tumor molecular characteristics, we can more effectively reveal the heterogeneity of RCC, and provide theoretical basis for the clinical diagnosis and prognosis of RCC. Finally, we developed a new scoring system based on six lncRNAs related to glycolysis aiming to accurately assess the prognosis of patients.

## Materials and Methods

### Collection of Transcriptional Data and Clinical Sample

RNA expression data and clinical information were downloaded from the [The Cancer Genome Atlas (TCGA)-GDC]^1^. The extracted clinical data included OS, age, sex, grade, stage, tumor size, distant metastasis, and lymph node metastasis. The glycolysis-related gene expression profiles downloaded from Gene Set Enrichment Analysis (GSEA)^2^. Patients with incomplete data or vague living status were excluded from our study. The clinical data were available in the database publicly, so this research required neither written approval from patients nor agreements from Ethics committee. All the data of this article was downloaded on October 15, 2020.

### Identification of Glycolysis-Related LncRNAs and Construction of Prognosis Model

Pearson correlation analysis was conducted to identify glycolysis-related lncRNAs. The correlation was calculated according to the expression value between lncRNAs and glycolysis genes. Our selection criteria were |R| > 0.4 and *P* < 0.001. Survival package of R was utilized for univariate and multivariate Cox regression analysis which provided hazard ratio (HR), β (cox) and *P*-values. The HRs were used to identify risk-related lncRNAs (HR > 1) and protective lncRNAs (HR < 1). Subsequently, we identified six target glycolysis-related lncRNAs for the prognostic signature model. Finally, the predictive model was constructed with six glycolysis-related lncRNAs through a previous formula ($\text{risk score}=\sum_{\left(i=1\right)}^{n}\text{Coef }\,\left(i\right)\times x\,\,\left(i\right)$) (Sun et al., 2020), where Coef (*i)* and *x* (*i*) represent the estimated regression coefficient and the expression value of each glycolysis-related lncRNAs, respectively.

### Evaluation and Verification of the Accuracy of Prognostic Signature

The Kaplan-Meier survival curve was utilized to show the survival correlation between high-risk group and low-risk group. Then, heatmap and scatter dot plot were used to observe the gene expression and prognosis of different groups. Finally, the receiver operating characteristic (ROC) curve was used to evaluate the accuracy of prediction. The correlation analysis was utilized to verify the connections between the risk score and clinicopathological characteristics of patients. We proved the independent prediction ability of glycolysis-related lncRNAs related prediction model through univariate and multivariate Cox regression analyses. In addition, stratified analysis was utilized to examine the precision of prognostic prediction in patient survival outcomes according to other clinicalpathological features.

### Establishment and Validation of Nomogram

A nomogram based on the risk scores and other clinicalpathological parameters was constructed to provide a reliable clinical prediction tool for RCC patients in 3- and 5-years survival time. Next, the calibration curves were applied to assess the concordance between predicted and actual observed patients. Finally, we also examined the area under the curve (AUC) values of ROC to determine the accuracy of our nomogram in predicting prognosis of patients.

### Gene Set Enrichment Analysis (GSEA)

In order to discover significant functional phenotype between high-risk group and low-risk group, we conducted GSEA. The GSEA software were downloaded from (see text footnote 2) and were conducted to distinguish differential expressed genes in high- and low-risk groups. The enriched gene sets were obtained based on a *P*-value < 0.05 and a false discovery rate (FDR) value < 0.25 after performing 1,000 permutations (Bandyopadhyay et al., 2014). Then the Kyoto Encyclopedia of Genes and Genomes (KEGG) pathway analysis was used to explore the exact signaling pathways related to high- and low-risk group.

### Construction of the LncRNA-mRNA Co-expression Network

The lncRNA-mRNA co-expression network was constructed using Cytoscape to research the correlation between the glycolysis-related lncRNAs and their target mRNAs and the potential functions of the six glycolysis-related lncRNAs in renal carcinoma.

### Statistical Analysis and Software Support

The data was processed via PERL programming langue^3^, Version (strawberry-perl-5.32.0.1-64bit.msi). Statistical analysis and figure constructing were conducted with R software^4^, version R x64 4.0.2.

## Results

### Acquisition of Prognostically Significant Glycolysis-Related LncRNAs in RCC Patient Tissue Samples

**FIGURE 1:**
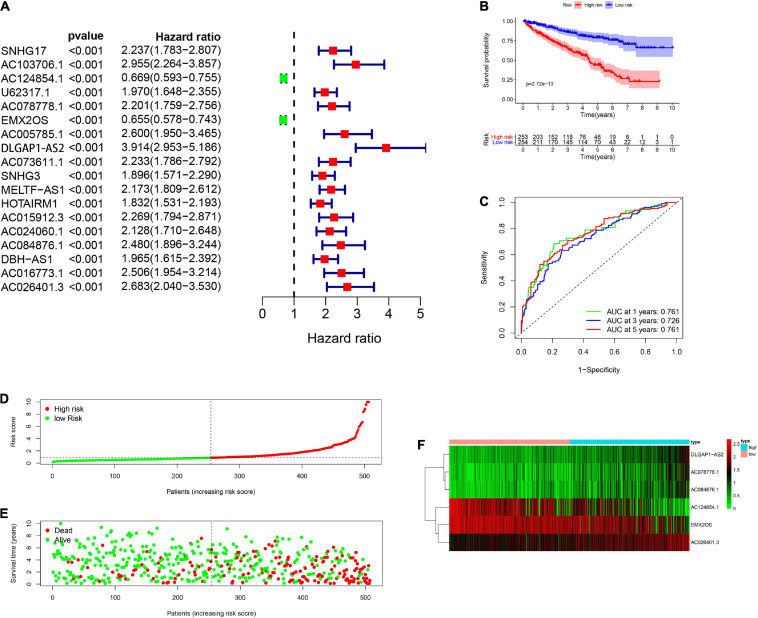
Construction and verification of glycolysis-related lncRNAs prognostic signatures in RCC patients. **(A)** The univariate Cox regression analysis combining the clinical surviving statistics exhibit that expression of 18 lncRNAs significantly correlated with OS of RCC patients. **(B)** Kaplan-Meier survival curve analysis shows that the significantly different survival time between the low-risk and the high-risk group. **(C)** ROC curve indicate the reliability of applying the risk score to predict the prognosis of RCC patients. **(D)** Distribution of high-risk and low-risk patients with renal cell carcinoma based on the glycolysis-related lncRNAs prognosis signature. **(E)** The scatter dot plot shows that the relationship between survival rates of the RCC patients and the risk score. **(F)** The heatmap shows the different expression of prognostic signature-related lncRNAs in different risk groups.

A total of 539 renal cancer patients were included for the subsequent study and the clinical information of 537 patients with renal clear cell carcinoma was downloaded at the same time. The complete clinical characteristics of the patients were included in Table 1. We also extracted glycolysis-related genes from the GSEA (see text footnote 2) and next acquired 1199 glycolysis-related lncRNAs by performing Pearson correlation analysis between the lncRNAs samples and the glycolysis-related genes setting | R| > 0.4 and *P* < 0.001 as the selection criteria. Univariate Cox regression analysis combining the clinical surviving statistics exhibited that expression of 18 lncRNAs significantly correlated with the survival time of RCC patients (*P* < 0.05; Figure 1A). Multivariate Cox regression analysis filtered 6 well candidate lncRNAs for constructing the prognostic signature (AC124854.1, AC078778.1, EMX2OS, DLGAP1-AS2, AC084876.1, and AC026401.3). Among the 6 lncRNAs, DLGAP1-AS2, AC084876.1, and AC026401.3 possessed the HR larger than 1 while the others smaller than 1 (Table 2).

**TABLE 1:** Summary of baseline clinical characteristics of patients with renal cancer.

Characteristic	TCGA
Age (years)	
≤65	340
>65	174
Gender	
Male	335
Female	179
Grade	
Low	230
High	276
T stage	
T1	263
T2	66
T3	75
T4	11
N stage	
N0	226
N1-3	17
AJCC stage	
I–II	310
III–IV	201
Survival status	
Alive	348
Deceased	166

**TABLE 2:** The information of target lncRNAs from the multivariate cox regression analysis.

Gene	Ensemble ID	Location	β(cox)	HR	*P*
AC124854.1	ENSG00000249776	chr5:92,410,256-92,671,581	−0.216164701	0.805603	0.006005
AC078778.1	ENSG00000258344	chr12:54,276,631-54,345,083	−0.388143004	0.678315	0.1036
EMX2OS	ENSG00000229847	chr10:117,470,801-117,545,068	−0.19260207	0.82481	0.020958
DLGAP1-AS2	ENSG00000262001	chr18:3,603,000-3,610,089	0.862480852	2.369031	0.000234
AC084876.1	ENSG00000277299	chr12:109,948,389-109,949,029	0.550076597	1.733386	0.010455
AC026401.3	ENSG00000280206	chr16:15,701,237-15,702,118	0.303059387	1.353995	0.074331

### Verifies the Accuracy of Six Glycolysis-Related LncRNAs Prognostic Signature

Then, the clinical patients were distinguished into two groups which included high-risk group (*n* = 253) and low-risk group (*n* = 254) according to their corresponding median cut off value. Kaplan-Meier survival curve analysis revealed that patients in the low-risk group presented a significant higher survival time and a better prognosis than those in the high-risk group (Figure 1B). ROC curve indicated the reliability of applying the risk scores to predict the prognosis of RCC patients in 1-, 3- and 5-years and all the AUC (area under the ROC) value greater than 0.7 (Figure 1C). RCC patients were then ranked according to the risk scores based on the glycolysis-related lncRNAs prognosis signature (Figure 1D). The scatter dot plot showed that the relationship between survival rates of the RCC patients and the risk score; patients with a higher risk score demonstrated lower survival time (Figure 1E). The heatmap showed the different expression of prognostic signature-related lncRNAs in the low-risk group and high-risk group. The risk factors (DLGAP1-AS2, AC084876.1, and AC078778.1) presented higher level in high-risk patients while low-risk patients presented higher levels of protective factors (AC124854.1, DLGAP1-AS2, and AC026401.3) (Figure 1F).

### Correlation Analysis of the Glycolysis-Related LncRNAs Prognosis Signature With Clinical Features

**FIGURE 2:**
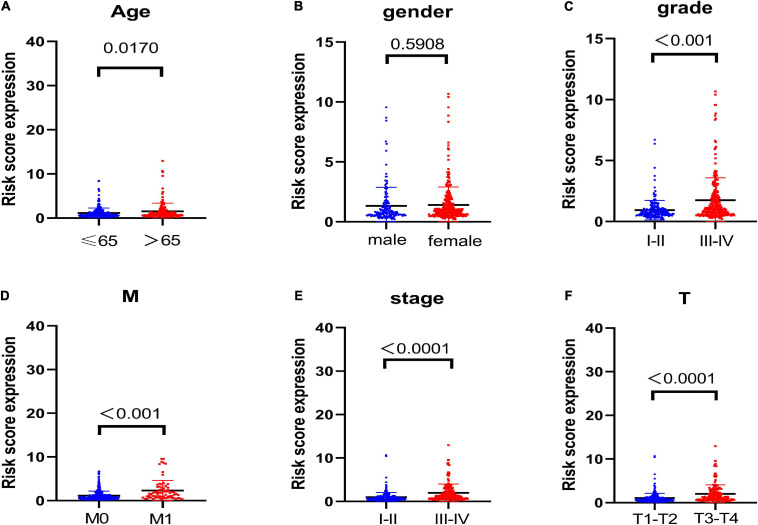
Correlation analysis of the glycolysis-related lncRNAs prognosis signature with clinical features. The results display that except for **(B)** gender (*P* = 0.5908), other parameters including **(A)** age (*P* = 0.017), **(C)** grade (*P* < 0.001), **(D)** M stage (*P* < 0.001), **(E)** AJCC stage (*P* < 0.0001), and **(F)** T stage (*P* < 0.001) are significantly related to risk score.

To discover the connections between the risk scores and clinicopathological characteristics of patients, we then analyzed the correlation and the results displayed that except for gender (Figure 2B, *P* = 0.5908), other parameters including age (Figure 2A, *P* = 0.017), grade (Figure 2C, *P* < 0.001), M stage (Figure 2D, *P* < 0.001), American Joint Committee on Cancer (AJCC), stage (Figure 2E, *P* < 0.0001), and T stage (Figure 2F, *P* < 0.001) were significant to risk scores. These results demonstrated that glycolysis-related lncRNAs risk signature was significantly related to renal carcinoma progression.

### Assess the Possibility of Independent Prediction of Glycolysis-Related LncRNAs Risk Signature

**FIGURE 3:**
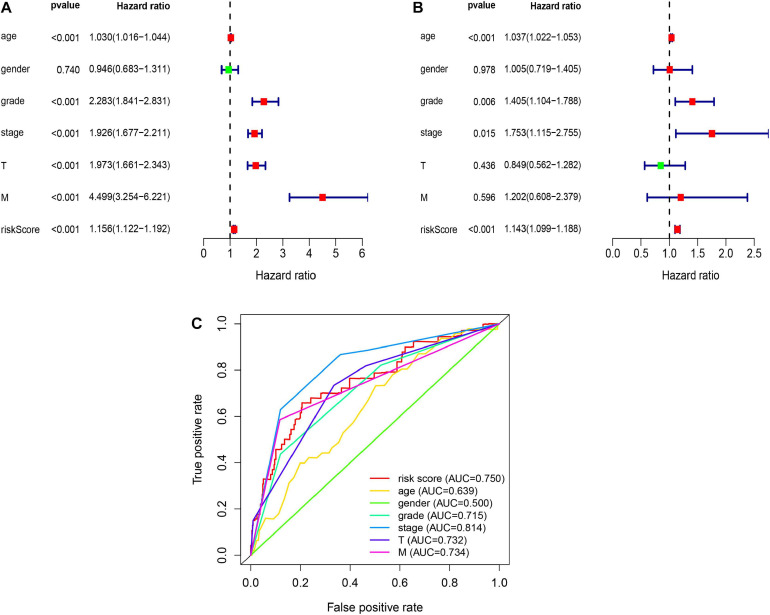
The independent prediction of glycolysis-related lncRNAs risk signature. **(A)** Univariate analysis reveal that glycolysis-related lncRNAs prognostic risk scores (*P* < 0.001) and other clinical features were significantly correlated with survival time except gender (*P* = 0.740). **(B)** Multivariate analysis shows that age (*P* < 0.001), AJCC stage (*P* < 0.05), grade (*P* < 0.05), and glycolysis-related lncRNAs prognostic risk score (*P* < 0.001) were independent prognostic indicators. **(C)** Multi-parameter ROC curve analysis shows the different AUC values among the clinicopathological characteristics.

Then, we verified whether the glycolysis-related lncRNAs prognostic signature was an independent prognostic factor in RCC patients by univariate and multivariate Cox regression analyses. Univariate analysis revealed that age (*P* < 0.001), AJCC stage (*P* < 0.001), grade (*P* < 0.001), T stage (*P* < 0.001), M stage (*P* < 0.001), and glycolysis-related lncRNAs prognostic risk score (*P* < 0.001) were significantly correlated with survival time except gender (*P* = 0.740) (Figure 3A). Multivariate analysis showed that age (*P* < 0.001), AJCC stage (*P* < 0.05), grade (*P* < 0.05), and glycolysis-related lncRNAs prognostic risk score (*P* < 0.001) were significant associated with survival time (Figure 3B). Meanwhile, Multi-parameter ROC curve analyses showed that the AUC value of glycolysis-related lncRNAs prognostic risk score was 0.750 (Figure 3C). All these statistical data demonstrated that the glycolysis-related lncRNAs risk score predict the prognosis independently in patients of RCC.

### Stratification Analysis According to Other Clinicalpathological Features

**FIGURE 4:**
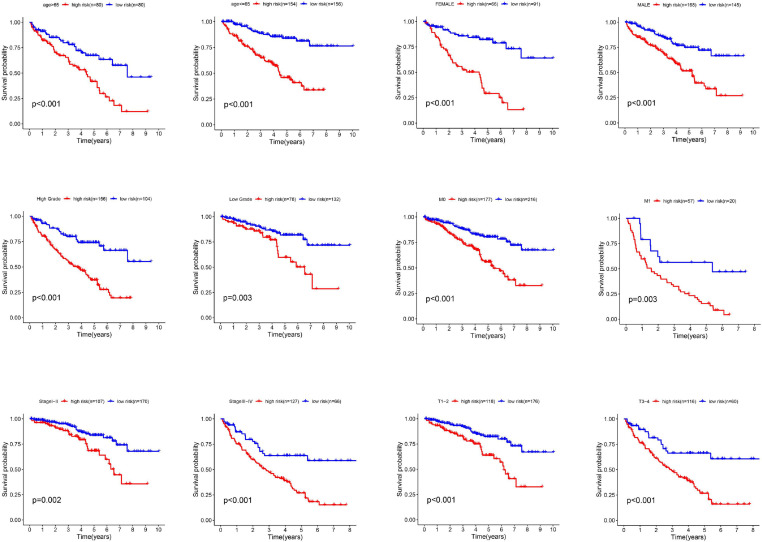
The different survival time of high- and low-risk groups classified by clinicopathological parameters. The Kaplan-Meier survival curve analysis shows that the correlation of risk score and OS among the patients with different characteristics such as age > 65 or age < 65, female or male, high grade or low grade, metastasis or non-metastasis, advanced-stage (Stage III–IV) or early stage (Stage I–II) and T1-2 or T3-4.

In the whole cohort, we performed a stratification analysis based on the clinicopathological characteristics of RCC patients (e.g., tumor stage, age, gender, and stage). As shown in Figure 4, the Kaplan-Meier survival curve analysis showed that glycolysis-related lncRNAs risk score was still significantly correlated with OS rate among the patients with age > 65 or age < 65, female or male, high grade or low grade, metastasis or non-metastasis, advanced-stage (Stage III–IV) or early stage (Stage I–II) and T1-2 or T3-4. All these results clarified that the risk score was still an accurate model for predicting prognosis in different patient groups.

### Quantification of Clinical Parameters and Evaluation of Prognostic Accuracy of Risk Score by Nomogram

**FIGURE 5:**
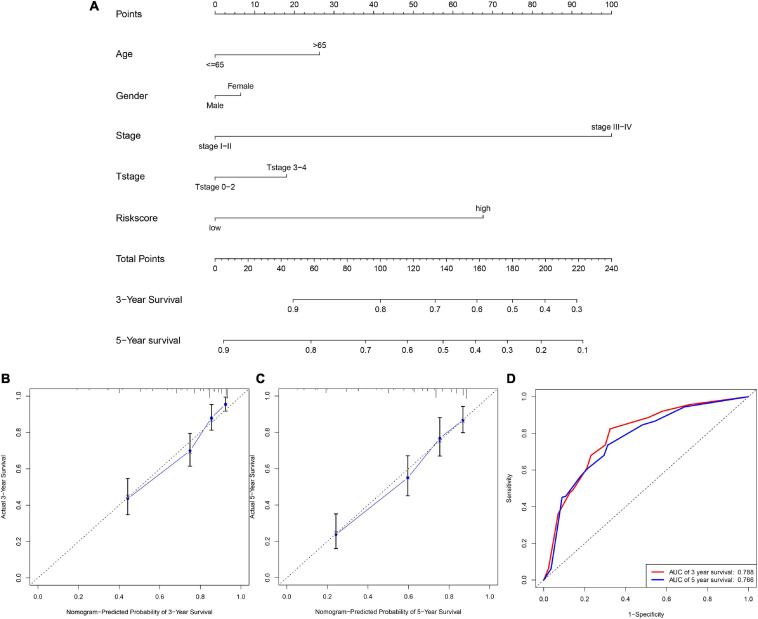
The nomogram based on clinicalpathological parameters and risk score. **(A)** The nomogram shows that the risk score accurately estimate the 3-, and 5-year survival time. **(B,C)** Calibration curves elucidate the concordance between predicted and observed 3- and 5-year OS of RCC patients. **(D)** ROC curve shows the AUC values (0.788 and 0.766) of 3- and 5-year survival.

In this section, the nomogram based on clinicalpathological parameters and glycolysis-related lncRNAs prognostic signature was applied to calculate the score for evaluating the precision of the prediction model. We constructed the nomogram (Figure 5A) combined multiple clinicalpathological characteristics including gender, age, T stage, AJCC stage, and the six glycolysis-related lncRNAs risk score to accurately estimate the 3-, and 5-year survival time by in RCC patients. The calibration curve analysis elucidated the concordance between predicted and actually observed 3- and 5-year OS of RCC patients (Figures 5B,C). Finally the time-dependent ROC (Figure 5D) showed that the AUC values of 3- and 5-year survival were 0.788 and 0.766, respectively. The above results verified that the risk score was reliable and accurate.

### Gene Set Enrichment Analysis (GSEA)

**FIGURE 6:**
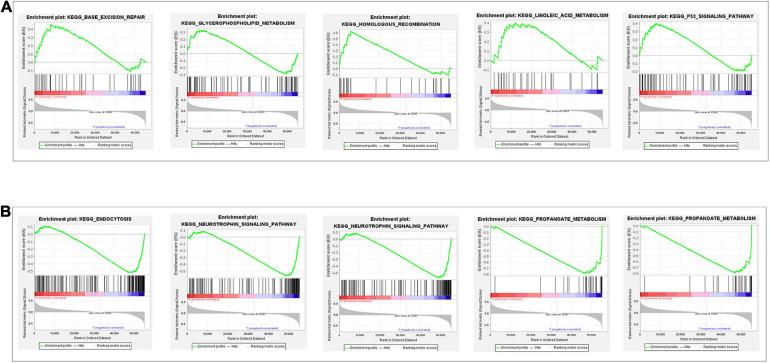
Gene set enrichment analysis of glycolysis-related gene sets between the high- and low-risk groups. **(A)** The results show significant enrichment of energy metabolism related signal pathway and cancer signal pathway in the high-risk group. **(B)** The results show immune related signal pathway and amino acid metabolism signal pathway in the low-risk group.

To depict the potential pathway and their corresponding functions of the glycolysis-related lncRNAs signature implicated in RCC progression, we performed GSEA between the high-risk group and low-risk group. The findings revealed that in the high-risk group, glycolysis-related lncRNAs signature exhibited a significant enrichment in energy metabolism related signal pathway and cancer signal pathway including base excision repair, glycerophospholipid metabolism, homologous recombination, linoleic acid metabolism, and P53 signaling pathway (Figure 6A). In addition, GSEA in low-risk group (Figure 6B) identified that immune related signal pathway and Amino acid metabolism signal Pathway such as endocytosis, neurotrophin signaling pathway, propanoate metabolism, tryptophan metabolism, valine leucine, and isoleucine degradation which suggested that the glycolysis-related lncRNAs signature participated in immune-related regulation. Our study provided some valuable insights for future investigations to discover new individualized treatments and achieve full-process management of RCC patients in different risk groups.

### Construction and Analysis of the LncRNA-mRNA Co-expression Network

**FIGURE 7:**
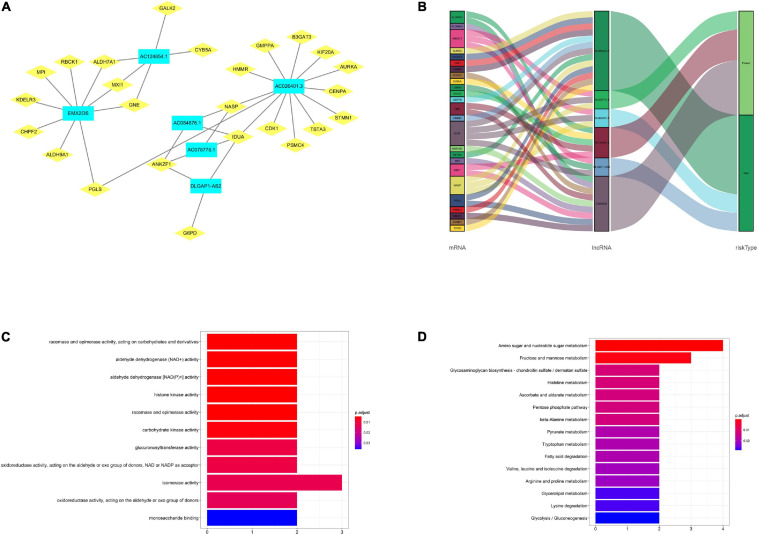
Construction and analysis of the lncRNA-mRNA co-expression network. **(A)** The diagram shows 36 lncRNA-mRNA links among 6 lncRNAs and 25 related mRNAs. **(B)** The Sankey diagram exhibited the correlation between the 25 mRNAs and 6 lncRNAs (risk/protective). **(C)** The GO term shows the enriched molecular biological metabolic processes. **(D)** The KEGG pathway analysis shows the significantly enriched pathways.

In our subject, the lncRNA-mRNA co-expression network was constructed using Cytoscape to research the potential functions of the six glycolysis-related lncRNAs in renal carcinoma. We found 36 lncRNA-mRNA links among 6 lncRNAs and 25 related mRNAs according to Pearson correlation coefficient |R| > 0.3 and *P* < 0.05 (Figure 7A). The Sankey diagram exhibited the correlation between the 25 mRNAs and 6 lncRNAs (risk/protective) (Figure 7B). According to these, 6 lncRNAs significantly correlated with the 25 mRNAs in the prognostic signature. Meanwhile, the top GO term for molecular biological metabolic processes were isomerase activity and racemase and epimerase activity, acting on carbohydrates and derivatives (Figure 7C). Finally, the KEGG pathway analysis showed that amino sugar and nucleotide sugar metabolism was the most significantly enriched pathway (Figure 7D).

## Discussion

Renal cell carcinoma is a typical heterogeneous malignant tumor with different subtypes and clinical manifestations in the genitourinary tumor family (Zhai et al., 2019; Patel et al., 2020). The treatment of RCC is mainly characterized by radical resection (Greef and Eisen, 2016) for insensitive to radiotherapy and chemotherapy and it is difficult for clinician to choose adjuvant therapy for post-operative recurrence. The biggest trouble faced by clinicians is how to identify high-risk patients early and make corresponding treatment strategies (Pierorazio et al., 2016). Because the prognosis of RCC is not positively related to the size of the tumor, the traditional TNM staging and clinical imaging data cannot meet this requirement (Lallas et al., 2015; Zabell et al., 2018). It is urgent to find new biomarkers to make molecular diagnosis and guide clinical strategies.

Previous studies have confirmed that lncRNAs as an important member of the non-coding RNA family, involved in the invasion and progression of RCC (Li D. et al., 2020). Shi et al. (2020) found that knocking down lncRNA DILA1 decreased Cyclin D1 protein expression and inhibited breast cancer cell growth both *in vitro* and *in vivo*. The lncRNA PVT1 is a novel oncogenic enhancer and its activity is controlled through aberrant methylation in colorectal cancer (Shigeyasu et al., 2020). MYC-mediated glycolysis of rectal tumor cells could be weakened with knocking out lncRNA LINRIS, which inhibited the progression of rectal cancer (Wang Y. et al., 2019). The glycolysis process was verified to be the energy source of tumor cell growth and invasion, which was regulated by downstream transcriptional activities mediated by lncRNA (Zhou et al., 2020). Glycolysis, as the main metabolic process in the cell metabolic microenvironment, participates in the proliferation of tumor cells. The glycolysis intermediates such as glucose 6-phosphate/pyruvate can participate in the regulation of fat and nucleic metabolism and other biological metabolic processes (Costanza et al., 2019; Orang et al., 2019; Smolle et al., 2020). In addition, the acidification of the microenvironment during glycolysis process is not conducive to the growth of normal cells, but is conducive to the infiltration and metastasis of tumor cells (Avella et al., 2020; Birts et al., 2020).

There are a lot of survival and prognosis analysis methods in the previous article. Some studies showed that “Gene mutations and copy number variants analysis” can be used to assess the difference between high- and low-risk groups (Xue et al., 2019). Some studies utilized logistic regression analysis and Cox proportional hazard analysis to verify the effect of prediction model (Kandimalla et al., 2020). In present study, we applied six glycolysis related lncRNAs (AC124854.1, AC078778.1, EMX2OS, DLGAP1-AS2, AC084876.1, and AC026401.3) which have not been previously reported to calculate the risk score, then confirmed the independent predictive ability by multivariate regression analysis with other clinical related parameters and verified the accuracy by ROC curve. Finally, the advantage of the predictive score of glycolysis related lncRNAs was shown in nomogram based on other clinical parameters (age, stage, T stage, etc.). GSEA enrichment analysis showed the enrichment of metabolic pathways in high- and low-risk patients, which further confirmed that glycolysis-related lncRNAs played a regulatory role in energy metabolism. High-risk group analysis indicated fat metabolism and tumor signal pathway enrichment; low-risk group analysis showed amino acid metabolism pathway enrichment, which also suggested that amino acid metabolism was involved in the occurrence of RCC and provided a potential target for the treatment of RCC in the future. It is generally believed that lncRNAs does not directly encode proteins, but through a variety of pathways to regulate gene expression, so as to promote tumorigenesis and tumor metastasis (Athie et al., 2020). Our study explored the lncRNA-mRNA regulatory network and described the biological function and signal pathway. There were many signal pathways about lncRNA-mRNA regulation, among which the classical tri-molecular regulatory network (lncRNA-miRNA-TF/gene) had been widely described in other malignant tumors (Ye et al., 2018; Li A. et al., 2020). By sharing the common miRNA-binding sites, lncRNA affected the function of downstream target genes and regulated the progress of the disease (Abdollahzadeh et al., 2019). In present study, GO term showed that the differential mRNA in the lncRNA-mRNA co-expression network mainly affected the biological functions such as isomerase activity. KEGG pathway enrichment analysis showed that the differential mRNA in the co-expression network was highly enriched in amino sugar and fructose and nucleotide sugar metabolism and mannose metabolism. However, these pathways were rarely studied in the pathogenesis of renal cancer cells, which provided a new target for the research of tumor progression.

Note that, some problems of distribution deviation have also attracted our attention in this research. Multivariate regression analysis showed that high-risk lncRNA (such as AC026401.3) was highly expressed in low-risk patients, while low-risk lncRNA (such as AC078778.1) showed high expression in high-risk patients. Previous reports have shown that tumor cells show a high degree of heterogeneity (Baliu-Piqué et al., 2020; Louault et al., 2020), and there may be individualized internal mechanisms or even opposite biological processes in different tumor stages. Therefore, we suspect that this inconsistent result may be related to the stage of tumor progression, and there would be different metabolic conditions between local and progressive tumor.

There are several limitations in our subject. First, the transcriptome sequencing data and clinical information downloaded from the public TCGA database which might be incomplete or biased in this research. Moreover, considering the lack of external sequence verification of other databases, it is need to prove the robustness of lncRNAs signature. Finally, our study is a theoretical research based on bio-informatics and statistical analysis, biochemical researches and animal experiments are required to confirm the results in further investigations.

## Conclusion

In summary, this study reveals that a glycolysis-based lncRNA signature (six candidate lncRNAs) exhibit diagnostic and prognostic values in RCC patients. Several related signaling pathway and metabolic relating activities may provide a potential target in clinical management and treatment of renal carcinoma. Since no relevant clinical data were found in other databases, we did not conduct external verification, which is indeed one of the shortcomings of this study. We will verify it in the clinical samples we collect later. At present, clinical patients are being followed up.

## Data Availability Statement

The original contributions presented in the study are included in the article/Supplementary Material, further inquiries can be directed to the corresponding author/s.

## Author Contributions

HC contributed to design the study and draft the manuscript. HT and JZ performed data acquisition and analysis. TL and ZQ performed the bioinformatics and the statistical data analysis. CX and XL constructed the figures and tables. WH was responsible for the integrity of the entire study and manuscript review. All authors read and approved the final manuscript.

## Conflict of Interest

The authors declare that the research was conducted in the absence of any commercial or financial relationships that could be construed as a potential conflict of interest.

## Footnotes

1 https://portal.gdc.cancer.gov/

2 http://www.gsea-msigdb.org/gsea/index.jsp

3 https://www.perl.org/

4 https://www.r-project.org/

## Abbreviations

AJCC: The American Joint Committee on Cancer
AUC: area under the curve
RCC: renal cell carcinoma
GO: Gene Ontology
GSEA: Gene Set Enrichment Analysis
HR: hazard ratio
KEGG: Kyoto Encyclopedia of Genes and Genomes
LncRNA: long non-coding RNA
OS: overall survival
ROC: receiver operating characteristic
TCGA: The Cancer Genome Atlas.
